# Association of thyroid hormone with osteoarthritis: from mendelian randomization and RNA sequencing analysis

**DOI:** 10.1186/s13018-024-04939-x

**Published:** 2024-07-25

**Authors:** Chengxin Li, Yucheng Tu, Rong Rong, Ziji Zhang, Weishen Chen, Lingli Long, Yangchun Zhang, Chao Wang, Baiqi Pan, Xiaoyu Wu, Mingqiang Guan, Bo Yang, Linli Zheng, Puyi Sheng

**Affiliations:** 1 Department of Joint Surgery, The First Affiliated Hospital of Sun Yat-Sen University, Zhong Shan 2nd Road, No. 58, Guangzhou, 510080 Guangdong China; 2 Department of Nosocomial Infection, The First Affiliated Hospital, Sun Yat-Sen University, Zhong Shan 2nd Road, No. 58, Guangzhou, 510080 Guangdong China; 3 Research Center of Translational Medicine, The First Affiliated Hospital of Sun Yat-Sen University, Zhong Shan 2nd Road, No. 58, Guangzhou, 510080 Guangdong China; 4 Department of Orthopedics, People’s Hospital of Shenzhen Baoan District, 118 Longjing 2nd Road, Shenzhen, 518101 Guangdong China; 5 Joint Surgery Center, Foshan Traditional Chinese Medicine Hospital, 6 Qinren Road, Foshan, 528200 Guangdong China; 6 Department of Orthopedics, The First Affiliated Hospital of Shantou University Medical College, 57 Chenghai Road, Shantou, 515041 Guangdong China

**Keywords:** Thyroid hormone levels, Osteoarthritis, Mendelian randomization, Hypertrophic chondrocyte, Triiodothyronine targeted genes, Diagnosis value

## Abstract

**Background:**

The relationship between thyroid hormone (TH) levels in vivo and osteoarthritis (OA) remains inconclusive. This study aims to investigate the association between TH levels and OA, analyze the effect of triiodothyronine on hypertrophic chondrocyte differentiation and OA progression, and identify potential target genes of triiodothyronine in OA to evaluate its diagnostic value.

**Methods:**

Two-sample mendelian randomization method was used to probe the causal links between hyperthyroidism and OA. Differentially expressed genes (DEGs) from two RNA-sequencing data in Gene Expression Omnibus (GSE199847 and GSE114007) and enrichment analysis of DEGs (166 commonly upregulated genes and 71 commonly downregulated genes of GSE199847 and GSE114007) was performed to analyze the effect of triiodothyronine (T3) on hypertrophic chondrocyte differentiation and OA. C28/I2 cells treated with T3 and reverse transcription and quantitative real-time polymerase chain reaction were used to validate T3 targeted genes. The diagnostic performance of target genes was assessed by the receiver operating characteristic (ROC) curve and area under the curve (AUC).

**Results:**

There was a positive causal association between hyperthyroidism and OA (IVW result, OR = 1.330, 95% CI 1.136–1.557, *P* = 0.0004). Weighted median and Weighted mode analysis also demonstrated that hyperthyroidism had a positive causal association with OA (*p* < 0.05, OR > 1). Bioinformatics analysis indicated T3 can partially induce the emergence of late hypertrophic chondrocyte and promote OA through extracellular matrix organization, blood vessel development, skeletal system development and ossification. Post-T3 treatment, MAFB, C1QTNF1, COL3A1 and ANGPTL2 were significantly elevated in C28/I2 cells. ROC curves in GSE114007 showed that AUC of all above genes were ≥ 0.7.

**Conclusions:**

This study identified that hyperthyroidism has a positive causal association with OA by MR analysis. T3 induced hypertrophic chondrocytes promote OA progression by upregulating genes such as MAFB, C1QTNF1, COL3A1 and ANGPTL2, which can also serve as OA diagnosis.

**Supplementary Information:**

The online version contains supplementary material available at 10.1186/s13018-024-04939-x.

## Introduction

Osteoarthritis (OA) is the most prevalent joint disorder, imposing a substantial burden on patients and society [1]. A worldwide estimate indicates that 10% of men and 18% of women aged 60 years or older have symptom [2, 3]. OA is characterized by the progressive degeneration of articular cartilage, along with changes in subchondral bone and other joint structures. Emerging evidence suggests that metabolic factors play a significant role in its pathogenesis [4]. However, the exact metabolic components that directly influence the development of OA are unclear. Recent years, the association between thyroid status and OA has attracted much attention [5, 6]. Nevertheless, research on the causal association and underlying mechanisms remains limited. Some researchers believe that elevated thyroid hormone (TH) levels can increase catabolism, promote apoptosis, and enhance pro-inflammatory responses, thereby exacerbating OA [7–9]. Houtman et al. [10] directly demonstrated that triiodothyronine (T3) can promote the transformation of chondrocyte towards hypertrophic phenotype. Existing studies also suggest that progression of OA is similar to endochondral ossification undergoing chondrocytes hypertrophy, autophagy, vascular invasion, and finally mineralization, which is precisely regulated by T3 and numerous growth factors [11–14]. Hypertrophic differentiation of chondrocytes is crucial in this process, how T3 regulates it in OA remains unclear, with few studies identifying specific target genes of T3. Therefore, this study aims to investigate the relationship between hyperthyroidism and OA through Mendelian randomization analysis, then analyze the effect of T3 on hypertrophic chondrocyte differentiation and OA, and finally identify the target genes of T3 in OA and evaluate its diagnostic value.

## Methods

### Data sources and mendelian randomization analysis

#### Data sources

The pooled data used to conduct this two-sample MR study were obtained from the IEU Open GWAS database summary website (https://gwas.mrcieu.ac.uk/). The exposure factors were hyperthyroidism (GWAS ID: ebi-a-GCST90038636), and the outcomes factor were OA (GWAS ID: ebi-a-GCST90038686), The above databases were derived from European or mixed populations. All datasets used in this study were from the public domain, and summary information is presented in Table 1.

**Table 1:** Summary of the GWAS included in this two-sample MR study

Variable	ID	Sample size	Number of SNPs	Population	Sex	Year
Hyperthyroidism or thyrotoxicosis	ebi-a-GCST90038636	484,598	9,587,836	NA	NA	2021
Osteoarthritis (OA)	ebi-a-GCST90038686	484,598	9,587,836	European	Males and Females	2021

#### Instrumental variable selection

Hyperthyroidism was used as the exposure factor, and single nucleotide polymorphisms (SNPs) significantly associated with this exposure were considered as instrumental variables (IVs). To ensure effective instrumental variables (IVs), the three fundamental assumptions of Mendelian randomization (MR) analysis must be satisfied. Firstly, the selected IVs must exhibit a robust correlation with the exposures. Secondly, there should be no confounding factors influencing the IVs that meet the exposure criteria. Thirdly, the selected IVs should solely impact the outcomes through the exposures. Initially, SNPs strongly associated with the exposure (*p* < 5 × 10^−8^) were identified. Subsequently, a manual screening process was conducted to eliminate SNPs linked to confounding factors and outcomes.

#### Mendelian randomization analysis

Causal association analysis between exposure and outcomes was conducted using a two-sample Mendelian randomization (MR) analysis approach based on a publicly available genome-wide association study (GWAS) database with large sample. We utilized the “Two Sample MR” R package (version 0.5.6, developed by Stephen Burgess, Chicago, IL, USA) to conduct two-sample Mendelian randomization (MR) analyses investigating the relationships between exposures and outcomes. The main analysis employed the inverse-variance weighted (IVW, random effects) method [15]. Additionally, supplementary analyses were performed using MR-Egger, weighted median, simple mode, and weighted mode methods [16]. The IVW method provides an accurate estimate when the assumption that all included single nucleotide polymorphisms (SNPs) can be utilized as effective instrumental variables (IVs) is met. MR-Egger regression is capable of detecting and adjusting for pleiotropy, albeit with lower estimation accuracy. The weighted median method offers an accurate estimate assuming that at least 50% of the IVs are valid. While not as powerful as IVW, the simple mode method provides robustness against pleiotropy. Weighted mode, however, is sensitive to the challenging selection of bandwidth for mode estimation.

#### Heterogeneity and sensitivity tests

Heterogeneity may arise in two-sample MR analyses due to variations in analysis platforms, experimental conditions (including SNPs), potentially leading to biased estimation of causal effects. Hence, this study assessed heterogeneity primarily using the main inverse-variance weighted (IVW) and MR-Egger methods [16]. The heterogeneity test evaluated differences among individual IVs, with Cochran’s Q statistic and associated P-value indicating heterogeneity (*P* < 0.1 denoting its presence). The pleiotropy test, crucial for detecting horizontal pleiotropy across multiple IVs, was also conducted. A P-value from this test was used to gauge the presence of pleiotropy in the analysis; if *P* > 0.05, it indicated weak likelihood of pleiotropy influencing the causal analysis. A leave-one-out sensitivity test was performed to ascertain the robustness of MR results [17]. By recalculating MR estimates after iteratively excluding individual IVs, any substantial deviation in the estimates signified sensitivity of the results to the excluded IV. Additionally, presence of pleiotropy was further evaluated using the MR-pleiotropy residual sum outlier (MRPRESSO) method.

### Data of RNA-sequencing and analysis

#### Data of RNA-sequencing

To identify DEGs in cartilage between OA patients and normals, as well as changes of gene expression in cartilage following thyroid hormone treatment, we search in GEO database with thyroid hormone, chondrocytes, osteoarthritis as key words. After excluding non-cartilage and non-human sequencing data, we obtained the gene expression profile of GSE199847 (Total RNA-sequencing in TC28a2 cell line with or without hypertrophic medium which consists of T3, ITS, BMP-2, GDF-5, Ascorbic acid, dexamethasone and β-glycerophosphate) and GSE114007(RNA-sequencing of 18 normal and 20 OA human knee cartilage tissues) from the Gene Expression Omnibus (GEO, https://www.ncbi.nlm.nih.gov/geo/) database for further analysis.

#### Bioinformatics analysis process and visualization

The R software DESeq2 package was used for analysis of differentially expressed genes (DEGs). In this analysis, |log2FoldChange|> 1 was set as the screening criterion for differences in expression, and p-adj < 0.05 was considered to indicate a significant difference. Subsequently, common DEGs were obtained by intersecting upregulated genes or downregulated genes between different RNA-sequencing data respectively. Volcano plot was drawn through the “ggplot2”package of R software. The Venn diagram was created using Adobe Illustrator 2022 after determining the number of DEGs. Finally, Gene Ontology (GO) analysis and Kyoto Encyclopedia of Genes and Genomes (KEGG) pathway enrichment of common DEGs were performed using Erichr online (https://maayanlab.cloud/Enrichr/) [18]. The top ten pathways with *P* < 0.05 were visualized.

### Cells and T3 treatment

The C28/I2 normal chondrogenic cell line was purchased from Sigma-Aldrich and cultured in DMEM/F-12 supplemented with 10% FBS (Gibco) and 1% penicillin–streptomycin. All cells were cultured at 37℃ in a humidified atmosphere consisting of 5% CO_2_ and 95% air. After the cells have reached confluence in the six-well plate, they were treated for 48 h with 10 ng/ml triiodothyronine (T3, Sigma-Aldrich), matching controls were left untreated.

### RNA isolation, reverse transcription and quantitative real-time polymerase chain reaction analysis

Total RNA from cells was extracted using TRIzol (Invitrogen, Carlsbad, CA, USA). cDNA was synthesized with the use of a reverse transcription kit (Toyobo, Osaka, Japan) and amplified using the SYBR Green Mix (Toyobo) on the Roche LightCycler 480 II (Roche, Basel, Switzerland). Quantitative real-time PCR (qRT-PCR) was conducted using EvaGreen Supermix (BIORad) on an iCycler Real-Time Detection System (BIORAD). GAPDH was used as an endogenous reference gene. Primer sequences are listed in Supplementary Table S1.

### Analysis of the diagnostic performance of target genes

The diagnostic performance of the target genes was evaluated using receiver operating characteristic (ROC) curve analysis, which plots sensitivity against specificity across different threshold values. The area under the ROC curve (AUC) was calculated to quantify the overall discriminatory ability of each gene as a diagnostic marker. According to the criterion if the AUC was ≥ 0.7 it was considered to have a clinical diagnostic value [19]. All target genes were subjected to ROC curves in GSE114007. The counts of target genes were extracted and placed in the Supplementary Table S5. ROC curve analysis and visualization were conducted using an open-source package available for R and S+ (https://www.bioinformatics.com.cn) [18].

## Results:

### Positive causal association between hyperthyroidism and OA identified by MR analysis

**Fig. 1:**
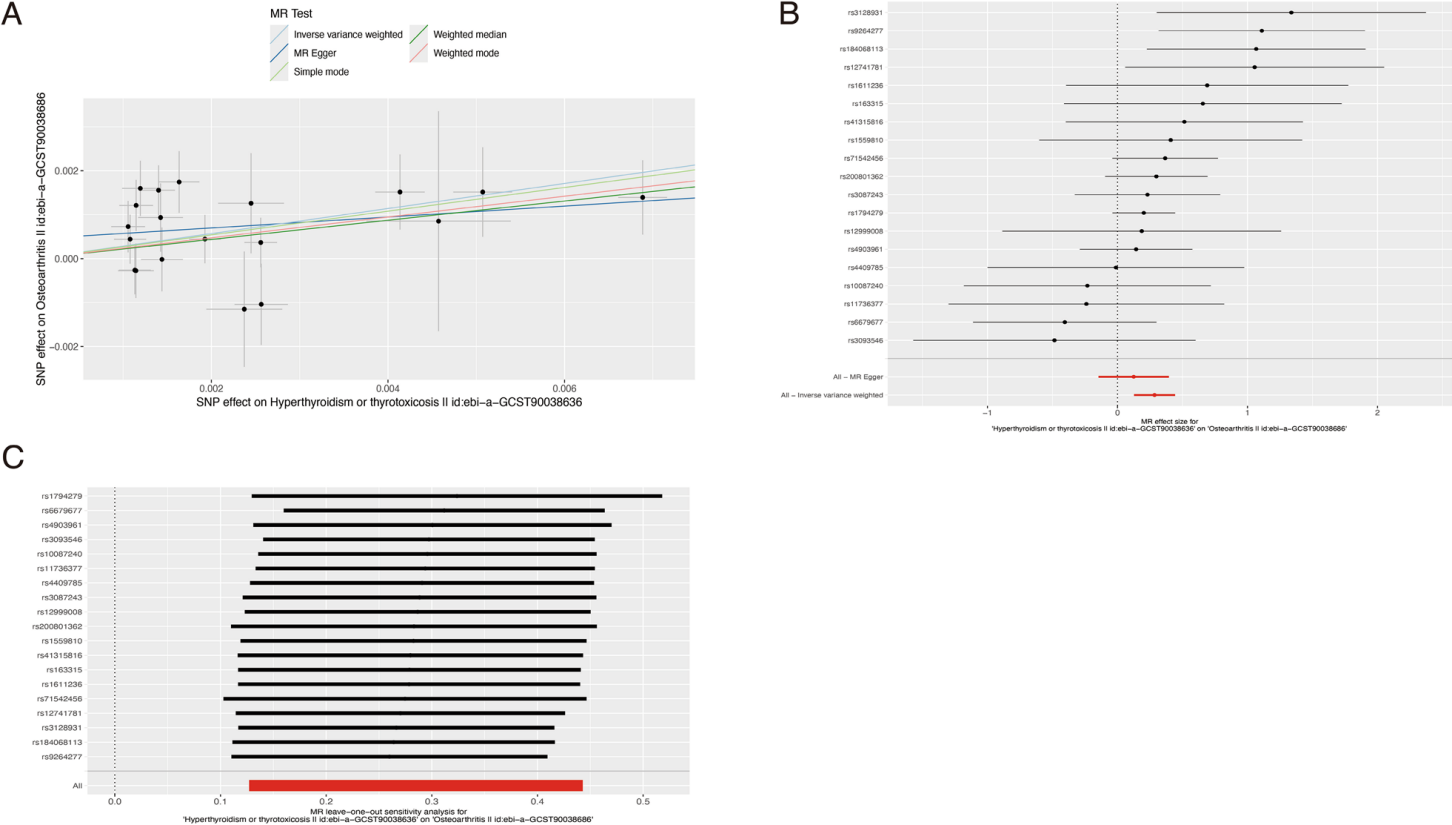
MR results of thyroxine levels and osteoarthritis (OA). **A** Scatter plot of genetic correlations of hyperthyroidism and OA using different MR methods. The slopes of line represent the causal effect of each method, respectively; **B** forest plot of the causal effects of hyperthyroidism associated SNPs on OA. The red and black dot/bar indicate the causal estimate of hyperthyroidism on risk of patients with OA; **C** forest plot of the “Leave-one-out”sensitivity analysis of hyperthyroidism associated SNPs on OA, indicating that no SNPs have a significant effect on the causal association estimates

After screening SNPs related to hyperthyroidism in the GWAS database and extracting the information of above SNPs corresponding to OA, we queried each SNP on the PhenoScanner website (http://www.phenoscanner.medschl.cam.ac.uk/) to exclude SNPs affected by confounding factors. We eventually obtained 19 SNPs as instrumental variables (IVs) for OA. IVW analysis showed that there was a positive causal association between hyperthyroidism and OA (OR = 1.330, 95% CI 1.136–1.557, *P* = 0.0004). Weighted median and Weighted mode analysis also demonstrated that hyperthyroidism had a positive causal association with OA (*p* < 0.05, OR > 1) (Table 2, Fig. 1A). IVW and MR-Egger test for heterogeneity showed that there was no heterogeneity in MR analysis results between hyperthyroidism and OA (all *p* > 0.05) (Table 3, Fig. 1B). The “Leave-one-out” sensitivity analysis also indicated that no SNPs have a significant effect on the causal association estimates (Fig. 1C).

**Table 2:** MR estimates of associations between hyperthyroidism and osteoarthritis(OA)

Exposure	Number of SNPs	MR methods	outcome	OR (95% CI)	*p* value
Hyperthyroidism or thyrotoxicosis	19	MR Egger	OA	1.132668775	0.379267068
		WME		1.244200059	0.018541911
		IVW		1.329755357	0.000403261
		Simple mode		1.309860245	0.106199639
		Weighted mode		1.267000197	0.017474458

**Table 3:** Results of heterogeneity and sensitivity test

Exposure	outcome	MR methods	p of pleiotropy	p of Cochran Q
Hyperthyroidism or thyrotoxicosis	OA		0.176	
		MR Egger		0.199
		IVW		0.149

### Exploring the impact of thyroid hormone on chondrocyte differentiation and osteoarthritis using bioinformatics analysis

In order to find the effect of thyroid hormone on chondrocyte differentiation and OA, we search in GEO database with "thyroid hormone", "chondrocytes" and "osteoarthritis" as key words. Lacking of sequencing data directly comparing the effects of thyroid hormone on cartilage differentiation, GSE199847 (Total RNA-sequencing in TC28a2 cell line with or without hypertrophic medium which consists of T3, ITS, BMP-2, GDF-5, Ascorbic acid, dexamethasone and β-glycerophosphate) and GSE114007(RNA-sequencing of 18 normal and 20 OA human knee cartilage tissues) were found for further analysis. A total of 2253 DEGs were found between OA and normal human cartilage (1311 upregulated and 942 down regulated), of which DIO2 and KLF17 were significantly upregulated (log2FC = 3.03, *P* = 2.41 × 10^–14^ and log2FC = 2.39, *P* = 1.5 × 10^–3^, respectively) (Supplementary Table S2, Figure S1). DIO2 regulates intracellular thyroid hormone levels by catalyzing the conversion of tetraiodothyronine (T4) to T3 (active form of T4)[20], while KLF9, as a downstream target of thyroid hormone signaling, is involved in metabolic regulation and developmental processes[21], indicating that the thyroid hormone pathway is activated in cartilage of OA. A total of 2736 DE genes were found between TC28a2 treated with hypertrophic media and DMEM (1668 upregulated and 1068 down regulated, adjusted *p* value < 0.05 and |log2FoldChange|> 1). Of these DEGs, THRA and KLF9 which suggest the activation of thyroid hormone pathway were once again found in the upregulated genes (log2FC = 1.27, *P* = 6.16 × 10–8 and log2FC = 1.64, *P* = 6.63 × 10–15, respectively). Some previously published early hypertrophic chondrocyte markers (RUNX2, COL10A1, MMP13, ALPL) were not found significantly upregulated, while mineralizing and late hypertrophy markers (EPAS1, COL1A1) were significantly upregulated by hypertrophic medium exposure [22, 23] (log2FC = 1.20, *P* = 2 × 10–4 and log2FC = 1.14, *P* = 2.30 × 10–4, respectively) (Supplementary Table S3, Figure S2). Together, these data suggest that thyroxine-related pathways are activated in both OA and hypertrophic differentiation. Moreover, hypertrophic medium containing T3 can induce the emergence of late hypertrophic chondrocyte that is inclined towards maturation and mineralization.

**Fig. 2:**
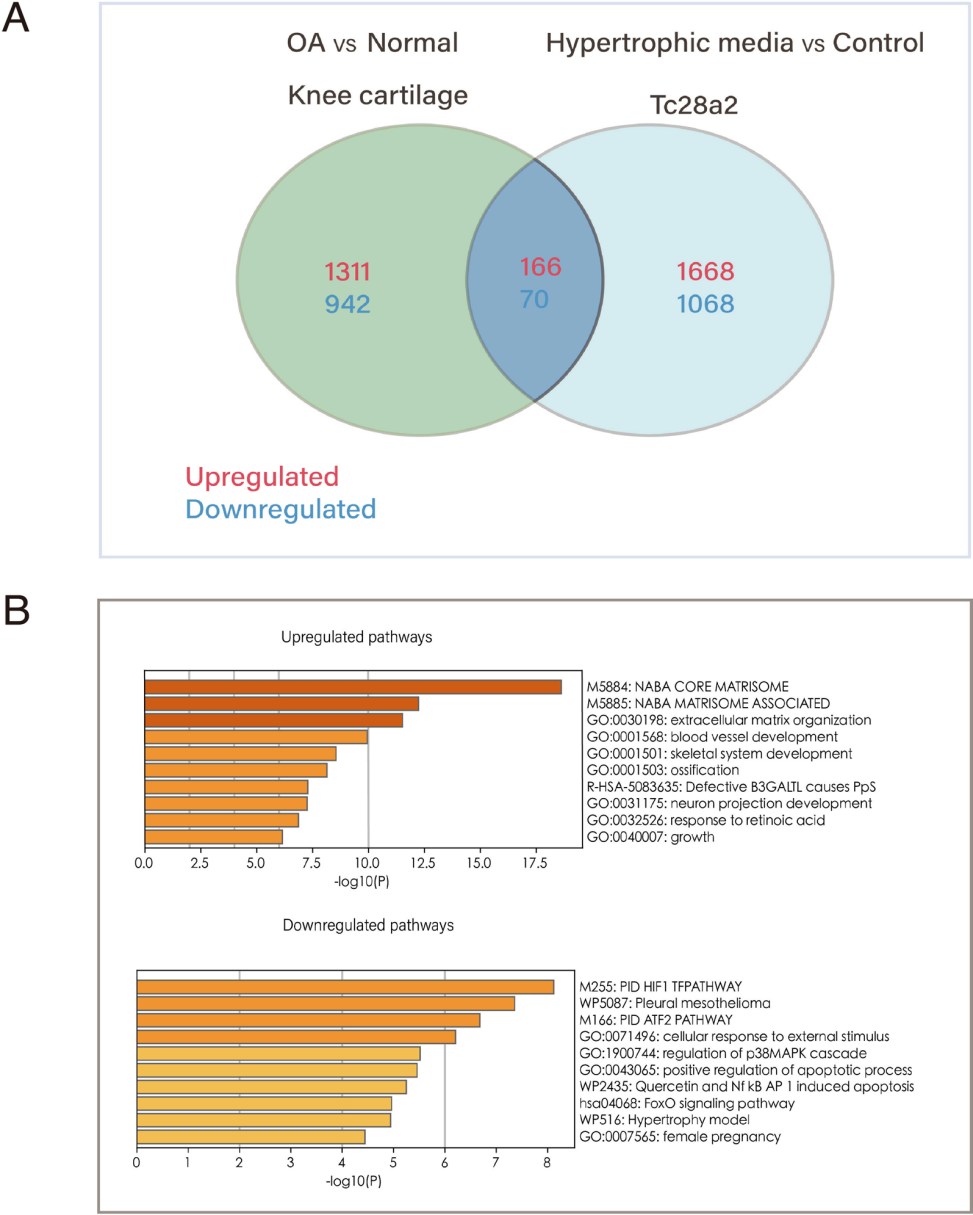
Relationship between chondrocytes induced by hypertrophic media and OA. **A** Venn diagram showing a total of 166 commonly upregulated genes and 71 commonly downregulated genes were identified. **B** KEGG-GO pathway analysis of common different expression genes

To further assess how hypertrophic medium with T3 influences OA progression, we intersected the DE genes from GSE114007 and GSE199847 and performed Gene Ontology (GO) and KEGG enrichment analysis of the upregulated and downregulated genes. A total of 166 commonly upregulated genes and 71 commonly downregulated genes were identified (Fig. 2A). GO and KEGG term analysis indicated an enrichment for upregulated genes encoding proteins mainly located in extracellular matrix organization, blood vessel development, skeletal system development and ossification. Downregulated genes were mainly enriched for processes involved in HIF-1, ATF2, cellular response to external stimulus, regulation of p38MAPK cascade and positive regulation of apoptotic process (Fig. 2B). These processes are in line with previous studies showing that chondrocytes in OA change into increased metabolic activity, angiogenesis inducing, and the recapitulation of endochondral ossification[24, 25].

### Bioinformatics analysis and experimental validation for identifying T3 target genes in OA and evaluating their diagnostic value

**Fig. 3:**
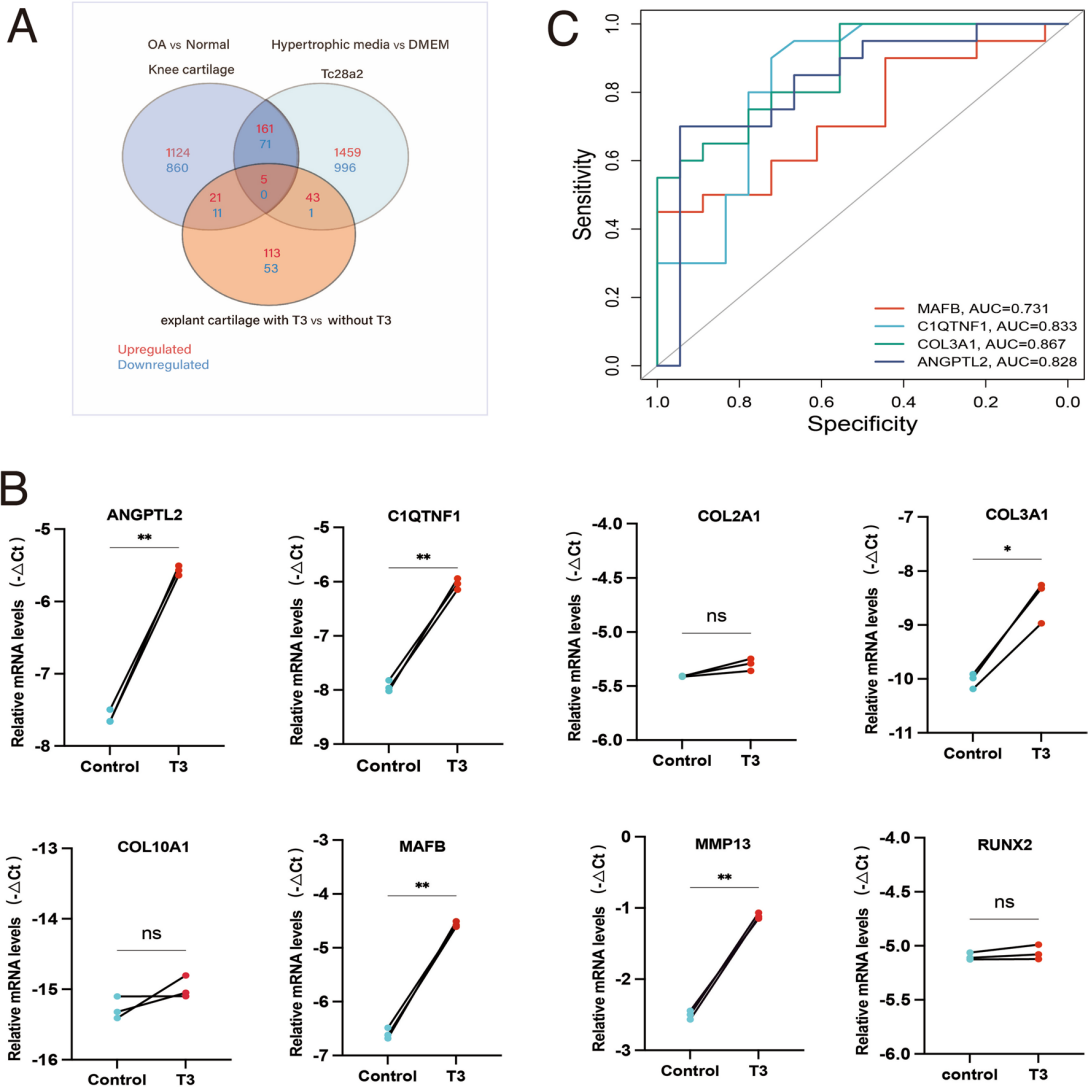
T3 target genes identification and diagnostic value exploration. **A** Venn diagram showing a total of 5 commonly upregulated genes and 0 commonly downregulated genes between T3 cartilage RNA-seq dataset, GSE114007 and GSE199847. **B** Gene expression in control and T3 stimulated C28/I2, figure shows connected paired samples. **C** The AUC of T3 targeted genes for OA diagnosis

In order to investigate the independent role of triiodothyronine (T3) in cartilage differentiation and osteoarthritis (OA), we intersected the DEGs from RNA-sequencing data of explant cartilage cultured ex vivo with or without T3)(Supplementary Table S4) with the DEGs identified from the two aforementioned sequencing datasets (GSE114007 and GSE199847).The results indicated that there were 5 commonly upregulated genes (MAFB,C1QTNF1,COL3A1,COL2A1,ANGPTL2), while no shared downregulated genes were found (Fig. 3A). Of these 5 upregulated genes, MAFB is the only transcript factor which can regulate chondrocyte differentiation[26]. C1QTNF1 and ANGPTL2 is known to have various biological functions, many of which are related to metabolism, inflammation and angiogenesis. COL3A1 and COL2A1 are crucial components of the extracellular matrix in cartilage. While COL3A1 is not as abundant in articular cartilage as COL2A1, it is involved in the early stages of wound healing and tissue repair. Overall, T3 leads to the upregulation of the aforementioned genes, reflecting a shift in cartilage metabolism from relatively quiescent to relatively active in OA.

To investigate the differences between T3 stimulated chondrocytes and classical hypertrophic chondrocytes, and to validate the aforementioned upregulated genes, we treated C28/I2 for 48 h with 10 ng/ml triiodothyronine (T3, Sigma-Aldrich). Then we examined the relative expression of traditional hypertrophic chondrocyte markers (COL10A1, MMP13, RUNX2) and the 5 genes above with qPCR. Post-T3 treatment, COL10A1, COL2A1 and the upstream transcription factor RUNX2 were not significantly upregulated. However, MMP13, MAFB, C1QTNF1, COL3A1 and ANGPTL2 were significantly elevated (Fig. 3B). In order to evaluate the effect of these new identified T3 targeted genes for OA diagnosis, MAFB, C1QTNF1, COL3A1 and ANGPTL2 were subjected to ROC curves in GSE114007. The AUC of all genes were ≥ 0.7 suggesting a clinical diagnostic value (Fig. 3C).

## Discussion

In this study, the causal relationship between hyperthyroidism and risk of osteoarthritis was investigated using a two-sample MR analysis method with publicly available databases and a large-scale GWAS study. Our results demonstrate that hyperthyroidism is associated with an increased risk of OA. Zhao et al.[27] also found that hyperthyroidism significantly increased the risk of OA using weighted multivariable-adjusted logistic regression (*P* = 0.018, odds ratio [OR] = 2.23, 95% confidence interval [CI] = 1.2–4.17). Their age-specific analysis further highlighted a stronger association in the 60–80-year-old age group (OR = 2.86, 95% CI = 1.46–5.59, *P* = 0.002). Results from Chen et al.[5] indicated a positive relationship between the total thyroxine relative index (TT4RI) and OA, where a higher TT4RI may indicate excess thyroxine (21.73 ± 19.28 for OA vs. 19.51 ± 19.14 for non-OA, *P* = 0.004). However, Tagoe et al.[28] found no significant association between anti-thyroid autoantibodies (TPOAb, TgAb) and radiographic knee osteoarthritis. Bos et al.[29] found that the enzyme DIO2, which promotes the conversion of local active thyroid hormone T3, is upregulated in OA patients. Pörings et al.[30] pointed out that the levels of T3 and its metabolites in the joint synovial fluid of OA patients are significantly higher than in their serum, indicating local thyroid hormone networks exist in the synovial fibroblasts of osteoarthritis patients. These results suggest that elevated TH level, especially local T3, is positively correlated with OA. Therefore, we cautiously conclude that hyperthyroidism may promote osteoarthritis through elevated thyroxine levels, highlighting the necessity for additional clinical and mechanistic studies.

T3 is essential for endochondral ossification in growth plate. During this process, chondrocytes undergo hypertrophy, autophagy, vascular invasion, and finally mineralization, which is precisely regulated by T3 and several paracrine factors [31, 32]. The progression of OA closely mirrors this process. Following hypertrophic changes in the articular chondrocytes, vascular invasion occurs, and ultimately, osteophyte formation is observed [14]. However, whether this process in osteoarthritis is also closely related to thyroid hormone remains unclear. Therefore, we hypothesize that thyroid hormone promotes OA progression by inducing hypertrophic differentiation and vascular invasion of articular chondrocytes.

Firstly, our bioinformatics analysis proved that the genes associated with the thyroxine (DIO2, KLF17) are significantly upregulated in OA cartilage, indicating that the thyroxine pathway is activated in OA. Existing research has demonstrated a close correlation between OA and deiodinase iodothyronine type 2 (DIO2), a type of intercellular T3 level regulation enzymes. Bomer et al. [33] found that increased DIO2 protein in OA-affected cartilage and allelic imbalance of OA risk polymorphism rs225014 at DIO2 in human OA joint tissues. Bomer et al. [34] also proved that DIO2^−/−^ mice are less prone to develop OA-like cartilage damage upon excessive mechanical stress and further pointed that DIO2 overexpression resulted in significant induction of ECM degrading enzymes (ADAMTS5, MMP13) and markers of mineralization (ALPL, COL1A1) through thyroid hormone signals. Houtman et al. [10] directly demonstrated that inhibit of the DIO2 activity by adding IOP to the osteochondral explants result in abolishment of MMP13 induction and COL2A1 reduction as well as a less hypertrophic environment as seen by a reduced COL10A1 expression. The above results demonstrate that increase of intracellular T3 can promote pathological changes in cartilage. The above findings validate our MR analysis results that hyperthyroidism is a risk factor for developing OA and suggest a novel therapeutic target for OA treatment. Therefore, our results provide scientific evidence for clinical interventions targeting thyroid hormone-related pathway and metabolism to prevent cartilage degeneration.

Secondly, our bioinformatics analysis suggested potential mechanisms and target genes through which thyroxine affects OA. Sequencing results indicated that classical hypertrophic markers such as RUNX2, COL10A1 and ALPL did not show significant upregulation. However, genes including MMP13, ADAMTS5, COL3A1, EPAS1 and COL1A1, were significantly elevated. These results suggest that the hypertrophic medium containing T3 alters the metabolic states and result in late hypertrophic differentiation of chondrocytes, which reported by Korthagen et al. [35] previously. Subsequent intersection and functional enrichment analyses of DEGs suggest that chondrocytes in the hypertrophic medium promote OA progression by affecting extracellular matrix organization, blood vessel development, skeletal system development and ossification. According to Guidotti et al. [36], adult chondrocytes and the extracellular cartilage matrix benefit from maintaining a low metabolic, maturation-arrested state. In contrast, chondrocytes in OA shift to increased metabolic activity, angiogenesis, and the recapitulation of endochondral ossification. Based on these findings, we propose that T3 partially contributes to a metabolically active, late-stage hypertrophic state in chondrocytes.

Since Houtman et al. [37] showed that staining with Safranin O confirmed that excess T3 was detrimental to cartilage homeostasis, as reflected by a loss of proteoglycans. Houtman et al. [38] also found that IOP (inhibition of intracellular active T3 production) reduced the detrimental changes of injurious mechanical stress, confirming the protective effect of blocking T3 production on cartilage, histologically. We intersected the DEGs of cartilage explants stimulated by T3 independently with the common DEGs from the previous analysis to further identify the target genes of T3 in OA from a mechanistic perspective. We found five commonly upregulated genes (MAFB, C1QTNF1, COL2A1, COL3A1 and ANGPTL2). Our experiments validated that T3 can not significantly upregulate COL10A1, a hypertrophic marker, or its upstream transcription factor RUNX2 in C28/I2 cells. However, MMP13, MAFB, C1QTNF1, COL3A1 and ANGPTL2 were significantly elevated. Notably, according to Korthagen et al. [35], upregulation of COL2A1 and COL3A1 by T3 is not generally beneficial but might be a response to injury that triggers an anabolic rescue response, that causes the chondrocytes to leave their maturely arrested state, leading to hypertrophy and the recuperation of a growth plate phenotype. C1QTNF1 and ANGPTL2 are two new genes qualified by qPCR that can be upregulated by T3. C1QTNF1 is a member of the C1q/TNF-related protein family playing an important role in regulating body energy homeostasis and the expression of proinflammatory cytokines [39]. ANGPTL2 is a protein that is known to promote inflammation and tissue remodeling. In OA cartilage, ANGPTL2 levels are typically elevated, which correlates with increased cartilage destruction and disease progression. Tanoue et al. [40] proved that ANGPTL2 contributes to chondrocyte differentiation and subsequent endochondral ossification during bone growth. MAFB, as a transcription factor, can regulate cell differentiation. Previous studies have shown that the genetic knockdown of MAFB in chondrocytes using siRNA (MAFB Si chondrocytes) abrogated the increased matrix metalloproteinase (MMP3 and MMP13) gene expression in rheumatoid arthritis [41]. These results support a role for MAFB as a regulator of chondrocyte gene expression and matrix formation via control of MMP3, and MMP13 expression, and indicate an important role for MAFB in chondrocytes. Combining our experimental results, we speculate that MAFB in cartilage can synergize with T3 to promote MMP13 expression and may be a key transcription factor in OA research. Finally, we performed ROC analysis on the aforementioned genes to determine their diagnostic value for OA. We found that MAFB, C1QTNF1, COL3A1 and ANGPTL2 could serve as potential markers for OA diagnosis (AUC > 0.7), with COL3A1 having the highest diagnostic sensitivity and specificity. Therefore, we consider the aforementioned four genes to be important targets of thyroxine and potential markers for OA diagnosis.

## Limitations

Firstly, in the Mendelian analysis, the data related to hyperthyroidism does not completely align with elevated thyroxine levels. Therefore, our results need to be interpreted cautiously. Secondly, our research lacks histopathological changes of cartilage direct exposing to thyroid hormone.

## Conclusion

In conclusion, this study identified that hyperthyroidism has a positive causal association with OA by MR analysis. Thyroxine induced hypertrophic chondrocytes promote OA through extracellular matrix organization, blood vessel development, skeletal system development, and ossification. T3 can promote the progression of OA by upregulating genes such as MAFB, C1QTNF1, COL3A1, and ANGPTL2, which can also serve as OA diagnosis.

### Supplementary Information

Below is the link to the electronic supplementary material.

Supplementary Material 1.

Supplementary Material 2. Figure S1. DEGs between OA and normal human cartilage.

Supplementary Material 3. Figure S2. DEGs between TC28a2 treated with hypertrophic media and DMEM.

## Data Availability

The datasets [Exposure/Outcome] for MR analysis can be found in the [IEU Open GWAS] [https://gwas.mrcieu.ac.uk/]. GSE114007 and GSE199847 can be found in the Gene Expression Omnibus (GEO, https://www.ncbi.nlm.nih.gov/geo/) database. DEGs after T3 treatment of osteochondral explants are available at https://doi.org/10.1186/s13075-024-03326-5. No datasets were generated or analysed during the current study.

## Abbreviations

ALPL: Alkaline phosphatase
AUC: Area under the curve
ATF2: Activating transcription factor 2
ANGPTL2: Angiopoietin like 2
C1QTNF1: C1q and TNF related 1
COL3A1: Collagen III, alpha 1
COL1A1: Collagen I, alpha 1
COL10A1: Collagen X, alpha 1
DEGs: Differentially expressed genes
DIO2: Deiodinase, Iodothyronine, Type II
EPAS1: Endothelial PAS domain-containing protein 1
FC: Fold change
GO: Gene ontology
GEO: Gene expression omnibus
GWAS: Genome-wide association study
HIF-1: Hypoxia-inducible factor 1
IVW: Inverse variance weighted
KEGG: Kyoto encyclopedia of genes and genomes
KLF9: Krueppel-like factor 9
KLF17: Krueppel-like factor 17
MR: Mendelian randomization
MAFB: MAF BZIP transcription factor B
MMP13: Matrix Metalloproteinase 13
MMP3: Matrix Metalloproteinase 3
OA: Osteoarthritis
ROC: Receiver operating characteristic
RUNX2: Runt-related transcription factor 2
RNA-seq: RNA sequencing
RT-qPCR: Reverse transcription quantitative polymerase chain reaction
SNP: Single nucleotide polymorphisms
T3: Triiodothyronine
T4: Tetraiodothyronine
THRA: Thyroid hormone receptor alpha
TH: Thyroid hormone
WME: Weighted median estimator
