# Supplementary figures and images for: Association of thyroid hormone with osteoarthritis: from mendelian randomization and RNA sequencing analysis
Source: J Orthop Surg Res. 2024 Jul 25;19:429. doi: 10.1186/s13018-024-04939-x (PMC11270794; doi:10.1186/s13018-024-04939-x)

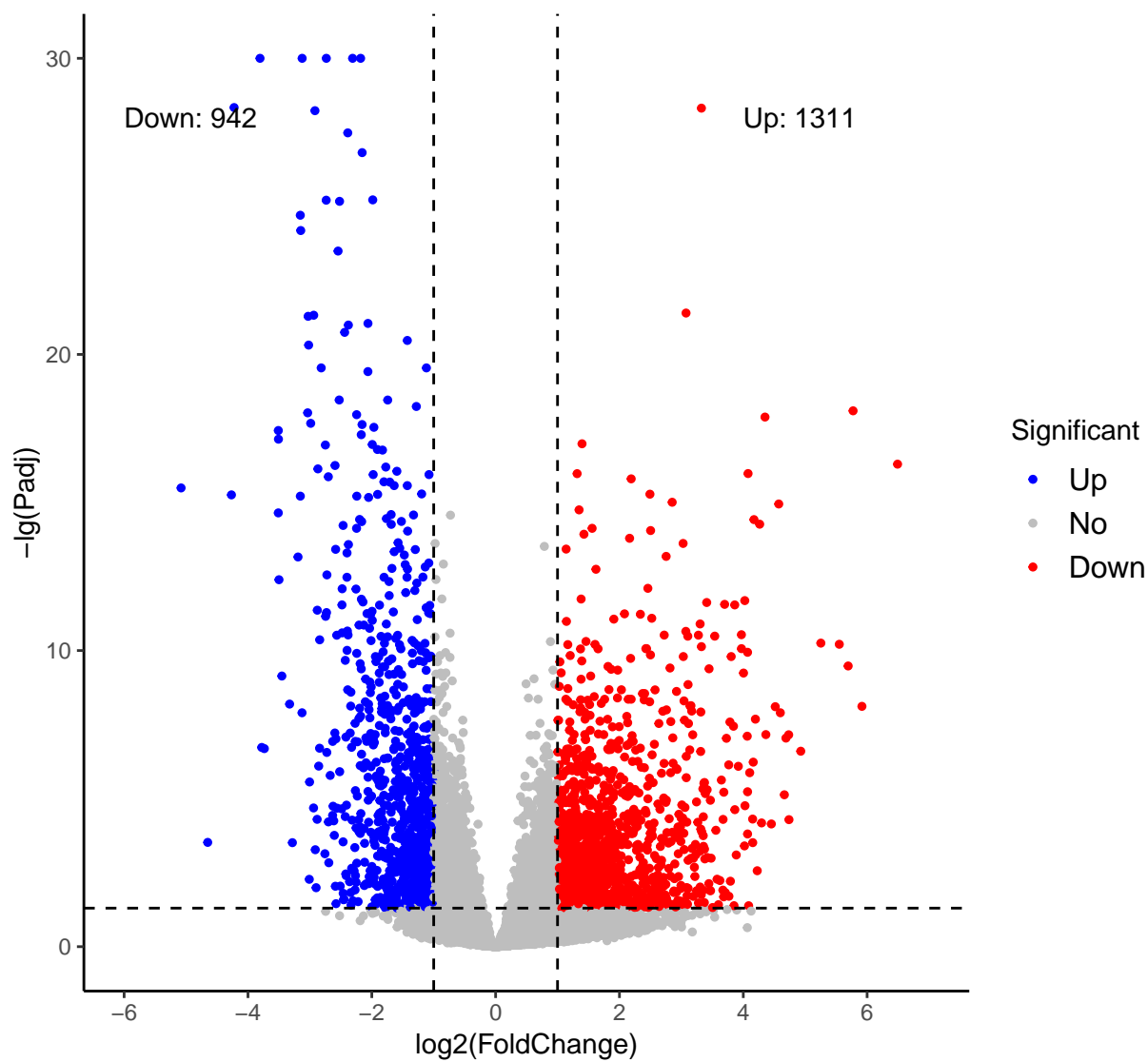

Supplement: Supplementary file 2 — Supplementary Material 2. Figure S1. DEGs between OA and normal human cartilage. [file 13018_2024_4939_MOESM2_ESM.pdf]

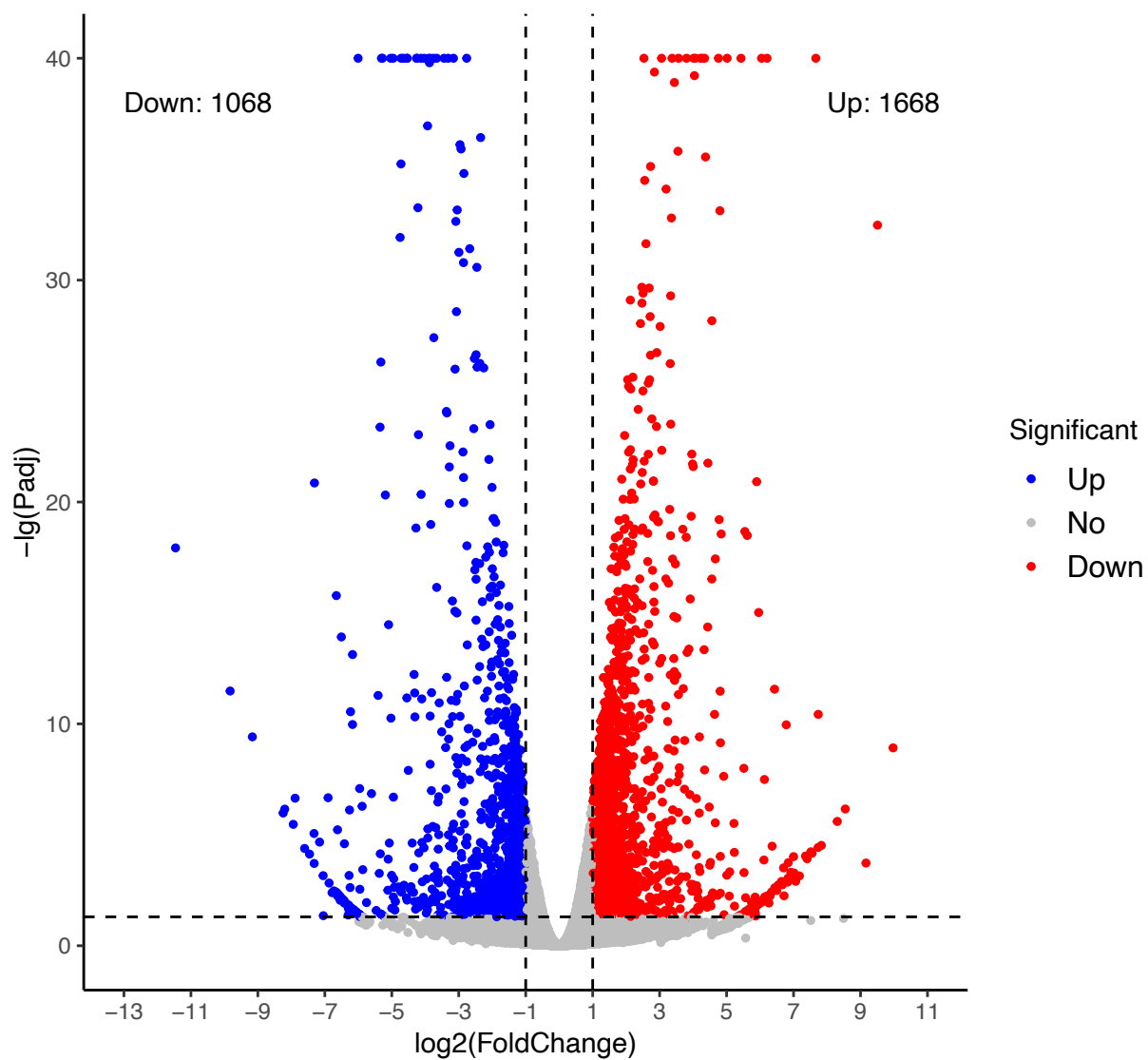

**Figure S2**

Supplement: Supplementary file 3 — Supplementary Material 3. Figure S2. DEGs between TC28a2 treated with hypertrophic media and DMEM. [file 13018_2024_4939_MOESM3_ESM.pdf]
